# Dose conformity and falloff in single‐lesion intracranial SRS with DCA and VMAT methods

**DOI:** 10.1002/acm2.14415

**Published:** 2024-06-24

**Authors:** Vadim Y. Kuperman, Yücel Altundal, Sunil Kandel, Tamara N. Kouskoulas

**Affiliations:** ^1^ Florida Cancer Specialists & Research Institute Hudson Florida USA; ^2^ AdventHealth Daytona Beach Florida USA

**Keywords:** conformity index, dose conformity, planning target volume

## Abstract

**Background:**

Intracranial stereotactic radiosurgery (SRS) aims at achieving highly conformal dose distribution and, at the same time, attaining rapid dose falloff outside the treatment target. SRS is performed using different techniques including dynamic conformal arcs (DCA) and volumetric modulated arc therapy (VMAT).

**Purpose:**

In this study, we compare dose conformity and falloff in DCA and VMAT plans for SRS with a single target.

**Methods:**

To compare dose conformity in SRS plans, we employ a novel conformity index $CI_{d_{exp}}$, RTOG conformity index ($CI_{RTOG}$), and Riet‐Paddick conformity index ($CI_{RP}$). In addition, we use indices R50%, $V_{10Gy}$, and $V_{12Gy}$ to evaluate dose falloff. For each of the considered 118 cases of SRS, two plans were created using DCA and VMAT. A two‐tailed Student's *t*‐test was used to evaluate the difference between the employed indices for the DCA and VMAT plans.

**Results:**

The studied VMAT plans were characterized by higher dose conformity than the DCA plans. The differences between the conformity indices for the DCA plans and VMAT plans were statistically significant. The DCA plans had a smaller number of monitor units (MUs) and smaller indices *R*50%, *V*_10_ *_Gy_*, and *V*_12_ *_Gy_* than the VMAT plans. However, the differences between *R*50%, *V*_10_ *_Gy_*, and *V*_12_ *_Gy_* for the DCA and VMAT plans were not statistically significant.

**Conclusions:**

Although the studied VMAT plans had higher dose conformity, they also had larger MUs than the DCA plans. In terms of dose falloff characterized by parameters *R*50%, *V*_10_ *_Gy_*, and *V*_12_ *_Gy_*, DCA serves as a reasonable alternative to VMAT in the case of a single brain metastasis.

## 1 INTRODUCTION

In intracranial stereotactic radiosurgery (SRS), high radiation dose is delivered to a small volume (normally < 2−3 cm in greatest dimension) by using non‐coplanar beams or arcs. The planning target volume (PTV) in SRS is produced by expanding the gross tumor volume (GTV) with expansion margin ≤ 2 mm. Depending on the size of the tumor, employment of margins ≥ 2 mm can lead to a significantly increased rate of brain necrosis.^1^

It should be realized that high dose conformity in SRS is required to minimize the volume of normal brain receiving doses greater than the prescribed dose.^2^, ^3^ However, dose conformity is not the only factor which needs to be assessed for every SRS plan. Previous studies found a significant correlation between radiation‐induced brain necrosis and volume of the brain tissue receiving doses greater than some threshold dose (e.g., 10 or 12 Gy^4^, ^5^). Consequently, rapid falloff of the delivered dose from its high values in the PTV to much lower values away from the target is also required. The dose falloff is frequently assessed by using a gradient measure (*R*50%) defined as “the ratio of the volume of half the prescription isodose to the volume of the prescription isodose”.^6^

### 1.1 Dose conformity indices

It has long been recognized that evaluation of dose conformity can be performed with the help of the so‐called conformity indices (CI) (e.g., see refs. 2, 3, 7, 8, 9, 10, 11, 12, 13, 14, 15, 16, 17, 18, 19, 20, 21, 22). To compute these indices, several input parameters are generally required: (a) volume of the planning target; (b) volume of the structure $A_{D_{ref}}$ representing all tissues which receive doses equal to or exceeding an arbitrary reference dose *D_ref_*; (c) volume of the intersection of $A_{D_{ref}}$ and PTV $\left(PTV\cap A_{D_{ref}}\right)$; (d) volume of the union of $A_{D_{ref}}$ and PTV $\left(PTV\cup A_{D_{ref}}\right)$. Note that these parameters can be computed by using built‐in software tools present in all commercially available treatment planning systems (TPS).

Among indices employed for evaluation of dose conformity, two indices are used most frequently: RTOG conformity index (*CI_RTOG_*)^2^, ^3^ and Riet‐Paddick index (*CI_RP_*).^7^, ^10^ The RTOG index is given by the ratio of two volumes:

(1) $$CI_{RTOG}=\frac{V_{D_{ref}}}{V_{PTV}},$$

where $V_{D_{ref}}$ and *V_PTV_* denote volumes of $A_{D_{ref}}$ and PTV, respectively. The Riet‐Paddick index is defined as follows:

(2) $$CI_{RP}=\frac{V_{\mathrm{int}}^{2}}{V_{PTV}V_{D_{ref}}},$$

where *V*_int_ denotes volume of the $PTV\cap A_{D_{ref}}$.

**FIGURE 1:**
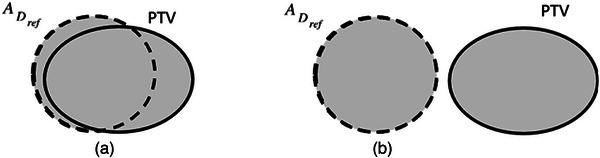
Schematic depiction of $A_{D_{ref}}$ and PTV. Unlike the case in (a), $A_{D_{ref}}$ and PTV do not intersect in (b).

**FIGURE 2:**
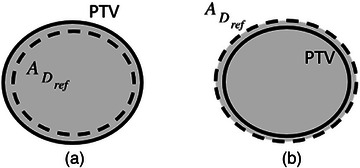
Two different scenarios describing underdosing the PTV and overdosing normal tissue are schematically shown in (a) and (b), respectively.

Both *CI_RTOG_* and *CI_RP_* have limitations which can be elucidated with the help of Figures 1 and 2. First, consider a diagram shown in Figure 1. If the volume of $V_{D_{ref}}$ and *V_PTV_* are fixed, then *CI_RTOG_* in (a) is equal to that in (b). However, it is clear that the conformity in (a) is higher than that in (b) because structures $A_{D_{ref}}$ and PTV in the latter case do not intersect.

Second, consider two different cases in Figure 2. Let us assume that *V*_int_ and the product $V_{PTV}V_{D_{ref}}$ are fixed. As a result, *CI_RP_* in (a) is equal to that in (b). Note that cases (a) and (b) in Figure 2 describe underdosing the PTV and overdosing normal tissue, respectively. Thus, one can conclude that *CI_RP_* does not differentiate between these scenarios.

Another feature of both *CI_RTOG_* and *CI_RP_* is their dependence on the PTV volume. As it was previously observed in ref. 9, in the case of a small target typically encountered in SRS, a relatively small change in the size of the target could result in a large relative change in the corresponding CI.

To reduce dependence on *V_PTV_*, a new conformity index $CI_{d_{exp}}$ was introduced in ref. 22. This index is defined as follows:

(3) $$CI_{d_{exp}}=\frac{V_{uni}-V_{int}}{V_{PTV_{exp}}-V_{PTV}},$$

where *V_uni_* is the volume of the union of $A_{D_{ref}}$ and PTV, $V_{PTV_{exp}}$ is the volume of the structure produced by expanding PTV with expansion margin *d_exp_*. As shown in ref. 22, $CI_{d_{exp}}$ has two attractive features which differentiate it from the RTOG and Riet‐Paddick indices: (a) $CI_{d_{exp}}$ is less dependent on *V_PTV_* compared to *CI_RTOG_* and *CI_RP_*; (b) unlike *CI_RTOG_* and *CI_RP_*, $CI_{d_{exp}}$ differentiates between two different scenarios corresponding to overdosing normal tissue and underdosing the PTV (see Appendix A). Note that in the “ideal” case $V_{uni}=V_{int}=V_{PTV}$. As a result, $CI_{d_{exp}}$ equals zero. In contrast, the corresponding indices *CI_RTOG_* and *CI_RP_* in this case are equal to one. In this work, *CI_RTOG_*, *CI_RP_*, and $CI_{d_{exp}}$ were used to compare dose conformity for SRS plans with DCA and corresponding plans with VMAT.

### 1.2 Dose falloff

As mentioned previously, dose falloff outside the target can be assessed by using a gradient index *R*50%. Additionally, in several studies it was demonstrated that brain necrosis caused by SRS depends on *V*_10_ *_Gy_* and *V*_12_ *_Gy_* which denote volumes of the normal brain tissue receiving doses equal to or greater than 10 and 12 Gy, respectively. As a result, in our work we employ *R*50%, *V*_10_ *_Gy_*, and *V*_12_ *_Gy_* to characterize dose falloff in the studied DCA and VMAT plans.

### 1.3 Main objectives

Previous studies comparing DCA and VMAT plans for SRS utilized a relatively small number of cases which likely contributed to a number of contradictory conclusions in refs. 23, 24, 25, 26. In contrast, our study uses a relatively large number of cases which facilitates statistical analysis of the differences between indices characterizing dose conformity and falloff. The main objectives of this work are twofold. The first objective is to establish whether the considered CI and dose falloff significantly differ between DCA and VMAT plans for SRS. The second objective of this work is to assess whether dose conformity and dose falloff are correlated. The latter objective is achieved by analyzing correlations between different CI and the two parameters deemed to have a high predictive value for the incidence of brain necrosis, that is, *V*_10_ *_Gy_* and *V*_12_ *_Gy_* (e.g.,^4^, ^27^, ^28^, ^29^).

## 2 METHODS

The GTV for SRS was delineated on the T1‐weighted, post‐contrast MR images of the brain. The MR images were fused with CT images used for treatment planning. The utilized CT images had a slice thickness ≤ 1.25 mm. In each case, the PTV was obtained by expanding the GTV in three dimensions with uniform expansion margin between 1 and 2 mm.

Dose distributions in 118 cases of intracranial SRS for a single brain metastasis were evaluated. Prescription dose was chosen based on the ASTRO guidelines.^30^ Other criteria for plan acceptance included dose constraints for the irradiated normal structures.^31^, ^32^ The treatment plans were created in the Eclipse TPS (version 15.6, Varian Medical Systems, Inc., Palo Alto, CA, USA) by employing non‐coplanar arcs. 6 MV beams with and without a flattening filter (FF) were used for treatment in 33 and 85 cases, respectively. The same treatment regimen 20 Gy × 1 = 20 Gy was used in all considered plans.

In each case, two treatment plans were created by using DCA and VMAT. The arrangement of non‐coplanar arcs (i.e., table position, start and stop angle for each arc) and dose grid were the same in the corresponding DCA and VMAT plans. The analytical anisotropic algorithm (AAA) and a grid size of 1.25 mm were used for dose computation. Each dose distribution was normalized so that 95% of the PTV received the prescribed dose per institutional standard.

In this study, each pair of plans for the same clinical case utilized either beams with a FF or flattening‐filter‐free beams (FFF). For example, if the treatment plan was created with VMAT and FF (or FFF) beams, then the second (research) plan was created by using DCA and FF (or FFF) beams.

### 2.1 Computation of CIs

Although different choices of *D_ref_* were previously suggested (e.g., see refs. 33, 34), in this work *D_ref_* was equal to the prescribed dose as recommended by RTOG 90‐05.^2^, ^3^ As a result, the RTOG conformity index *CI_RTOG_* was given by the ratio of the volume encompassed by the prescribed isodose surface and volume of the PTV (see Equation 1). Computation of *CI_RTOG_* for each plan was performed by using a built‐in software tool in Eclipse.

The Riet‐Paddick index *CI_RP_* was computed with the help of the dose‐volume histogram (DVH) as follows. First, volume of the PTV receiving dose $D⩾D_{ref}$ was determined from the planned DVH. This volume was equal to *V*_int_. Second, volume of all tissue receiving doses equal to or greater than *D_ref_* (i.e., $V_{D_{ref}}$) was also determined with the help of DVH. Note that $V_{D_{ref}}$ is given by the sum of two terms:

(4) $$V_{D_{ref}}=V_{NT}+V_{int},$$

where *V_NT_* represents volume of the normal brain tissue receiving dose $D⩾D_{ref}$. Subsequently, *CI_RP_* was computed by using Equation (2).

To compute $CI_{d_{exp}}$ in Equation (3), one needs to compute *V_uni_* and $V_{PTV_{exp}}$. The former volume was computed by using the following relationship^35^:

(5) $$V_{uni}=V_{PTV}+V_{D_{ref}}-V_{int}.$$

Finally, $V_{PTV_{exp}}$ was determined by expanding PTV with uniform expansion margin *d_exp_* = 0.1 cm. As discussed previously in ref. 22, the choice of expansion margin is defined by the desired average distance between the reference isodose surface and the surface of the PTV. For example, if the desired average distance between the two surfaces is less than 0.1 cm, then *d_exp_* = 0.1 cm should be chosen. Because a high degree of dose conformity is expected for intracranial SRS, *d_exp_* of 0.1 cm is a suitable choice. For other treatment approaches and/or treatment sites, *d_exp_* of 0.2 cm might be a better option.

### 2.2 Computation of *R*50%, *V*_10Gy_, *V*_12Gy_ and HI

As mentioned previously, *R*50% is given by the ratio $\frac{V_{D_{ref/2}}}{V_{D_{ref}}}$, where $V_{D_{ref/2}}$ represents volume of all tissues receiving dose $D⩾\frac{D_{ref}}{2}$. On the other hand, *V*_10_ *_Gy_* and *V*_12_ *_Gy_* represent volumes of the normal brain tissue receiving doses equal to or greater than 10 and 12 Gy, respectively. Like $V_{D_{ref}}$, volumes $V_{D_{ref/2}},V_{10Gy},\;\mathrm{and}\;V_{12Gy}$ were determined by using DVH.

In addition to the above‐mentioned indices, we also computed homogeneity index (HI) given by the ratio $\frac{D_{max}-D_{min}}{\bar{D}}$, where $D_{max},D_{min},\mathrm{and}\;\bar{D}$ denote maximum, minimum, and average doses in the PTV, respectively.

### 2.3 Data analysis

The employed indices, their mean values and variances were computed in Microsoft Excel (Microsoft Corporation, 2024: https://office.microsoft.com/excel). A two‐tailed Student's *t*‐test was used to evaluate statistical significance of the differences between the indices computed for the DCA and VMAT plans. The *p*‐values were computed by using R software for data analysis (version 4.3.3, R Foundation for Statistical Computing, Vienna, Austria: https://www.R‐project.org).^36^ The Pearson correlation coefficient was used to characterize correlations between the utilized CIs, *V*_10Gy_, and *V*_12Gy_ (see Appendix B).

## 3 RESULTS

**FIGURE 3:**
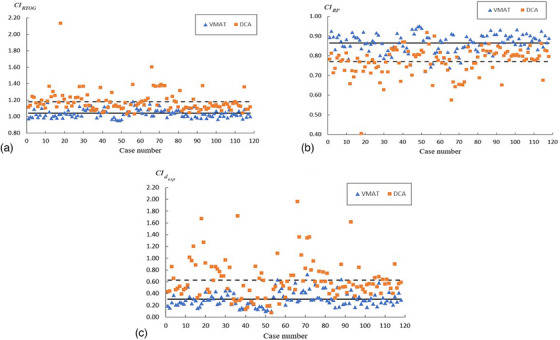
$CI_{RTOG},\;CI_{RP,}$ and $CI_{d_{exp}}$ for the considered DCA and VMAT plans. Dashed and solid lines in Figures 3 and 4 depict the sample means of the indices for the DCA and VMAT plans, respectively.

**FIGURE 4:**
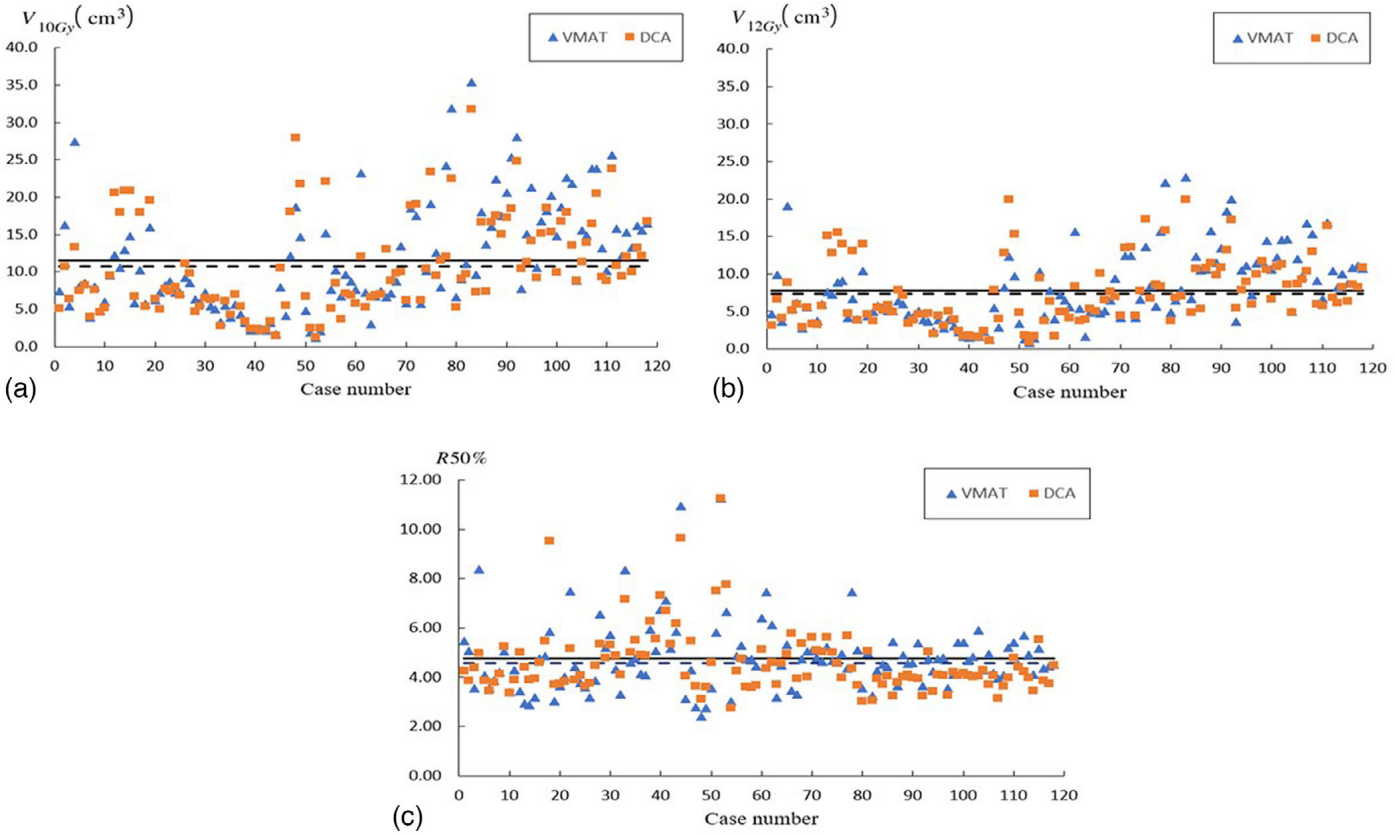
$R50\%,V_{10Gy\;}\mathrm{and}\;V_{12Gy}$ for the DCA and VMAT plans.

Table 1 contains locations of the intracranial lesions for the studied cases of SRS. Figure 3 displays *CI_RTOG_*, *CI_RP_*, and $CI_{d_{exp}}$ for the evaluated DCA and VMAT plans. Dosimetric indices characterizing dose falloff are shown in Figure 4. Table 2 contains the sample mean and sample standard deviation (STD) of the considered CIs, *R*50%, *V*_10_ *_Gy_*, and *V*_12_ *_Gy_*, *V_NT_*, HI, *D_min_*, and *D_max_*. Table 3 includes the computed sample correlation coefficients between the studied CIs and each of the following two volumes: *V*_10_ *_Gy_* and *V*_12_ *_Gy_*.

**TABLE 1:** Lesion locations and volumes for the studied cases of SRS.

Tumor location	Number of cases	*V_PTV,min_* (cm^3^)	*V_PTV,max_* (cm^3^)	*V_PTV,aver_* (cm^3^)
BS	2	7.0	10.8	8.9
LC	9	0.5	7.9	3.9
LF	15	0.6	13.0	5.3
LO	5	1.4	14.1	6.1
LP	15	1.5	11.9	5.6
LT	4	1.0	8.9	5.1
MC	5	1.7	9.4	5.3
RC	10	0.4	10.7	3.2
RF	22	1.5	8.8	4.5
RO	5	0.4	6.2	3.0
RP	14	0.2	8.9	3.2
RT	12	0.1	8.2	3.6

Abbreviations: BS, brainstem; LC, left cerebellum; LF, left frontal; LO, left occipital; LP, left parietal; LT, left temporal; MC, middle cerebellum; RC, right cerebellum; RF, right frontal; RO, right occipital; RP, right parietal; RT, right temporal.

**TABLE 2:** Dosimetric indices for the studied DCA and VMAT plans.

	DCA	VMAT	*p*‐value^*^
*CI_RTOG_*	1.18 ± 0.13	1.04 ± 0.05	<0.001
*CI_RP_*	0.77 ± 0.07	0.86 ± 0.05	<0.001
$CI_{d_{exp}}$	0.63 ± 0.33	0.31 ± 0.13	<0.001
*R50%*	4.58 ± 1.31	4.76 ± 1.41	0.306
*HI*	0.24 ± 0.10	0.15 ± 0.05	<0.001
$D_{min}\;\left(\mathrm{cGy}\right)$	1802.4 ± 267.2	1907.1 ± 262.6	0.003
$D_{max}\;\left(\mathrm{cGy}\right)$	2348.4 ± 268.6	2246.9 ± 219.5	0.002
MU	3098.0 ± 342.7	5078.1 ± 987.2	<0.001
*V*_12Gy_ (cm^3^)	7.34 ± 4.27	7.77 ± 4.79	0.474
*V*_10Gy_ (cm^3^)	10.75 ± 6.34	11.49 ± 7.13	0.402
*V_NT_* (cm^3^)	1.02 ± 0.82	0.36 ± 0.30	<0.001

*Note*: Each index is presented as $\bar{Index}\pm STD$, where Index and STD denote the sample mean and sample standard deviation, respectively.

* A *p*‐value less than 0.05 was considered statistically significant.

**TABLE 3:** Sample Pearson correlation coefficient *r* for several dosimetric indices.

Parameters	DCA	VMAT	Correlation strength^a^
*V_PTV_* and *V*_10Gy_	0.79	0.70	Strong
*V_PTV_* and *V*_12Gy_	0.76	0.69	Strong
*CI_RTOG_* and *V*_10Gy_	−0.06	−0.25	Weak
*CI_RTOG_* and *V*_12Gy_	−0.01	−0.22	Weak
*CI_RP_* and *V*_10Gy_	0.07	0.25	Weak
*CI_RP_* and *V*_12Gy_	0.02	0.22	Weak
$CI_{d_{exp}}\mathrm{and}\;V_{10\mathrm{Gy}}$	0.26	0.20	Weak
$CI_{d_{exp}}\mathrm{and}\;V_{12\mathrm{Gy}}$	0.29	0.24	Weak

^a^ Strong and weak correlations correspond to |*r*| > 0.6 and |*r*| < 0.3, respectively.

## 4 DISCUSSION

The focus of our study is on the comparison of dose conformity and dose falloff in treatment plans for intracranial SRS created by using DCA and VMAT. The relevant compared parameters included *CI_RTOG_*, *CI_RP_*, and $CI_{d_{exp}}$, gradient index *R*50% and three indices describing dose given to normal brain tissue, that is, *V*_10_ *_Gy_* and *V*_12_ *_Gy_*, and *V_NT_*. In addition, HI was also computed.

It should be mentioned that different CIs and HI for DCA plans in SRS were previously considered in refs. 37, 38, 39. In the framework of our work, the analysis of dosimetric indices in refs. 23, 24, 25, 26 is of particular interest, since these studies compared DCA and VMAT based plans for SRS.

Note that previous investigations have resulted in contradictory conclusions regarding compared indices for SRS. For example, based on analysis of 10 cases of SRS with a single lesion, Molinier et al.^23^ previously found that DCA plans were characterized by lower *V*_10_ *_Gy_* and, as a result, were better than the corresponding VMAT plans. In contrast, the opposite conclusion was reached by Torizuka et al.^24^; that is, for the analyzed 15 cases of SRS, *V*_10_ *_Gy_*, and *V*_12_ *_Gy_* were significantly smaller in the plans with VMAT than those observed in the DCA plans. In agreement with,^24^ Perez‐Calatayud et al.^25^ stated that for the analyzed five cases of SRS originally planned with DCA and replanned with VMAT, the latter approach resulted in a superior sparing of normal brain tissue manifested in lower values of *V*_10_ *_Gy_* and *V*_12_ *_Gy_* in all considered cases. Conversely, better sparing of normal brain tissue with DCA was noticed in ref. 26. The results of our study are summarized below.

### 4.1 *CI_RTOG_, CI_RP_* and $CI_{d_{exp}}$

The analysis of CIs (see Figure 3 and Table 2) indicates that dose conformity in the studied VMAT plans was higher than that in the DCA plans. The performed Student's *t*‐test confirmed that the differences between *CI_RTOG_*, *CI_RP_*, and $CI_{d_{exp}}$ for the VMAT plans and those for the DCA plans were statistically significant (i.e., *p*‐values were less than 0.05). It should be mentioned that despite higher dose conformity in VMAT plans, DCA method offers advantages due to simpler treatment planning and delivery, and fewer monitor units (MUs) (e.g., refs. 23, 24, 25).

As discussed previously, the standard indices *CI_RTOG_* and *CI_RP_* do not differentiate between two different scenarios, that is, overdosing normal tissue and underdosing PTV. In contrast, $CI_{d_{exp}}$ can be expressed as a sum of two terms which are proportional to *V_NT_* and volume of the PTV receiving dose less than the prescribed dose (see Appendix A). Because in this work the DCA and VMAT plans were normalized in such a way so that 95% of the PTV received the prescribed dose, smaller values of $CI_{d_{exp}}$ for VMAT plans indicate smaller values of *V_NT_* (see next Section).

It should be mentioned that $CI_{d_{exp}}$ appears to be noisier than the standard indices *CI_RTOG_* and *CI_RP_* (see Figure 3). The observed feature of $CI_{d_{exp}}$ is likely due to the fact that this index is more sensitive to small differences between $V_{D_{ref}}$ and *V_PTV_* than the standard indices (see Appendix C).

Finally, it should be realized that the novel index $CI_{d_{exp}}$ has several limitations. The first limitation of $CI_{d_{exp}}$ (as well as limitation of the standard indices *CI_RTOG_* and *CI_RP_*) is associated with a highly irregular shape of the treatment target. In this case, condition $CI_{d_{exp}}<\;1$ which signifies acceptable dose non‐conformity,^22^ can conceal local mismatch between the shapes of $A_{D_{ref}}$ and PTV. The second limitation of $CI_{d_{exp}}$ is due to the size of the dose grid which impacts accuracy of computed *V_uni_* and *V_int_* in Equation (3). Evaluation of the latter limitation indicates that a uniform dose grid ≤1.25mm is desirable for accurate computation of $CI_{d_{exp}}$ in the case of small target volumes typical for SRS.^22^

### 4.2 *R*50%, *V*_10_ *_Gy_*, *V*_12_ *_Gy_* and *V_NT_*

Compared to the VMAT plans, smaller mean values of *R*50%, *V*_10_ *_Gy_*, and *V*_12_ *_Gy_* were observed in the DCA plans (see Figure 4 and Table 2). However, the differences between these parameters in the studied DCA and VMAT plans were not statistically significant, that is, *p* > 0.05 (see Table 2). Note also that VMAT plans were characterized by smaller values of *V_NT_*. The difference between the sample means of *V_NT_* for VMAT and DCA was statistically significant.

### 4.3 MUs, *D*_min_, *D*_max_ and HI

In addition to the parameters discussed in Sections 4.1 and 4.2, Table 2 also includes the sample means of MUs, homogeneity index HI, *D_min_*, and *D_max_*. It should be mentioned that the studied VMAT plans had greater MUs, greater *D_min_* and smaller *D_max_* than the corresponding DCA plans. The differences between MUs, *D_min_*, and *D_max_* in the VMAT and DCA plans were statistically significant. Compared to the plans with VMAT, smaller MUs in the plans with DCA lead to shorter beam‐on times and, as a result, help reduce patient motion during treatment.

Note that HI was smaller for the VMAT plans compared to that for DCA plans. The difference between the sample means of HI for VMAT and DCA plans was statistically significant.

### 4.4 Interpretation of the results

The findings of this work indicate higher dose conformity, lower dose inhomogeneity in the target and lower *V_NT_* in VMAT plans compared to those in DCA plans. Although it is logical to expect that higher dose conformity correlates with better treatment outcome in SRS, a recent analysis of 925 cases with a single brain metastasis,^37^ concluded that *lower* dose conformity in SRS for a single metastatic tumor was associated with a *lower* rate of local progression of the disease. In addition, the same study concluded that gradient index *R*50% had no impact on complication rates. Conversely, a number of previous studies^4^, ^38^, ^39^, ^40^ found a significant correlation between *V*_10Gy_ or *V*_12Gy_ and the radiation‐induced brain necrosis in patients treated with intracranial SRS.

To establish correlation between different dosimetric indices, we also computed the sample Pearson correlation coefficient *r* ^41^ for several pairs of the studied indices. Strong positive correlation (i.e., *r* > 0.6) was found between *V_PTV_* and *V*_10Gy_, and between $V_{PTV}\;\mathrm{and}\;V_{12\mathrm{Gy}}$ (see Table 3). Conversely, weak correlation (i.e., |r|<0.3) was found between $V_{10Gy},V_{12\mathrm{Gy}}$ and CIs. These results indicate that, in contrast to $V_{PTV}$, the considered CIs do not serve as a good predictor of brain necrosis for SRS cases planned with DCA or VMAT.

It should be mentioned that the obtained results can be used to establish an acceptable range for variations of dosimetric indices in intracranial SRS. For example, if one assumes Gaussian distribution for each CI, then about 95% of the computed CIs will fall within 2 STD from the population mean. By replacing the population mean and population STD with the sample mean ($\bar{CI}$) and sample STD, respectively, the range of expected variations can be defined as $\left[\bar{CI}-2\cdot STD,\;\bar{CI}+2\cdot STD\right]$.

## 5 CONCLUSIONS

The results indicate that dose conformity in VMAT plans is higher than that in DCA plans for SRS; however, this fact alone does not indicate superiority of VMAT method over DCA in SRS. More importantly, the studied CIs did not display strong correlations with $V_{10\mathrm{Gy}}\;\mathrm{and}\;V_{12\mathrm{Gy}}$ used as predictors of radiation‐induced brain necrosis.

Compared to the VMAT plans, the studied DCA plans were characterized by smaller mean values of R50%, $V_{10\mathrm{Gy}},\;V_{12\mathrm{Gy}}$, and smaller MUs. The results indicate that in terms of these parameters, DCA remains a reasonable alternative to VMAT in the case of a single brain metastasis.

## AUTHOR CONTRIBUTIONS

*Design of the study, collection, analysis and interpretation of data, drafting the manuscript, revising and approving its final version submitted for publication*: Vadim Y. Kuperman. *Collection, analysis and interpretation of data, revising and approving the final version of the work submitted for publication*: Yücel Altundal. *Collection and analysis of data, revising and approving it for the final version submitted for publication*: Sunil Kandel. *Collection and analysis of data, revising and approving it for the final version submitted for publication*: Tamara N. Kouskoulas.

## CONFLICT OF INTEREST STATEMENT

The authors declare no conflicts of interest.

## APPENDIX A

### A.1

Expression for conformity index $CI_{d_{exp}}$ in Equation (3) can be written as follows:

(A1)
$$CI_{d_{exp}}=\frac{V_{uni}-V_{PTV}}{V_{PTV_{exp}}-V_{PTV}}+\frac{V_{PTV}-V_{int}}{V_{PTV_{exp}}-V_{PTV}}.$$

By using Equations (4) and (5), we obtain

(A2)
$$V_{uni}=V_{PTV}+V_{NT}$$

As a result, $CI_{d_{exp}}$ can be expressed as

(A3)
$$CI_{d_{exp}}=\frac{V_{NT}}{V_{PTV_{exp}}-V_{PTV}}+\frac{V_{PTV}-V_{int}}{V_{PTV_{exp}}-V_{PTV}}.$$

Equation (A3) indicates that in the case when compared plans have the same dose coverage of the PTV, the difference between the corresponding indices $CI_{d_{exp}}$ is proportional to the difference in the volumes of the normal tissue receiving the prescribed dose in the compared plans.

To elucidate the properties of $CI_{d_{exp}}$, it is instructive to consider this index in the cases shown in Figure 2. In the case from Figure 2a we have $V_{NT}=0\;\mathrm{and}\;CI_{d_{exp}}$ is proportional to the fraction of the PTV which is underdosed. In contrast, in the case from Figure 2b we have $V_{PTV}-V_{int}=0\;\mathrm{and}\;CI_{d_{exp}}$ is proportional to the volume of the normal tissue receiving doses greater than or equal to the prescribed dose.

## APPENDIX B

### Pearson correlation coefficient

Consider a pair of random variables *X* and *Y* with samples $\left(x_{1},..,x_{n}\right)\;\mathrm{and}\;\left(y_{1},..,y_{n}\right)$, respectively. The sample Pearson correlation coefficient r is defined as follows (e.g., ref. 41):

(B1)
$$r=\frac{\sum_{i=1}^{n}\left(x_{i}-\bar{X}\right)\left(y_{i}-\bar{Y}\right)}{\sqrt{\sum_{i=1}^{n}{\left(x_{i}-\bar{X}\right)}^{2}}\sqrt{\sum_{i=1}^{n}{\left(y_{i}-\bar{Y}\right)}^{2}}},$$

where $\bar{X}\;\mathrm{and}\;\bar{Y}$ denote the sample means of *X* and *Y*, respectively.

## APPENDIX C

### C.1

Suppose that PTV and $A_{D_{ref}}$ are spheres with the same center. Let *r* and r+δr denote radii of the PTV and $A_{D_{ref}}$, respectively. We have

(C1)
$$\begin{array}{ccc} V_{PTV} & = & \frac{4\pi r^{3}}{3}\;\mathrm{and}\;V_{D_{ref}}=\frac{4\pi {\left(r+\delta r\right)}^{3}}{3}\\ & & =\frac{4\pi }{3}\left(r^{3}+3r^{2}\delta r+3r\delta r^{2}+\delta r^{3}\right). \end{array}$$

In the case δr≪r, we obtain from (C1)

(C2)
$$CI_{RTOG}=\frac{V_{D_{ref}}}{V_{PTV}}\approx \frac{r^{3}+3r^{2}\delta r}{r^{3}}=1+\frac{3\delta r}{r}$$

and

(C3)
$$CI_{RP}=\frac{V_{PTV}}{V_{D_{ref}}}\approx \frac{r^{3}}{r^{3}+3r^{2}\delta r}=1-\frac{3\delta r}{r}.$$

It is straightforward to show that

(C4)
$$CI_{d_{exp}}=\frac{V_{uni}-V_{int}}{V_{PTV_{exp}-}V_{PTV}}=\frac{r^{2}\delta r+r\delta r^{2}+\frac{\delta r^{3}}{3}}{r^{2}d_{exp}+rd_{exp}^{2}+\frac{d_{exp}^{3}}{3}}.$$

In the case when both $d_{exp}\;\mathrm{and}\;\delta r\;\ll \;r$, Equation (C4) yields

(C5)
$$CI_{d_{exp}}\approx \frac{\delta r}{d_{exp}}.$$

Equatoin (C5) indicates that for $CI_{d_{exp}}$ as a function of δr, the rate of change equals $1/d_{exp}$. Conversely, for indices $CI_{RTOG}$ and $CI_{RP}$, the rates of change are proportional to 3/r. Consequently, in the case $r>3d_{exp}$, the same change in the average separation between $A_{D_{ref}}$ and PTV (i.e., the same δr) leads to a greater change in $CI_{d_{exp}}$ as compared to the corresponding changes in $CI_{RTOG}$ and $CI_{RP}$.
